# Sensory Deficits in Mice with Lateral Spinal Cord Hemisection Mimic the Brown–Séquard Syndrome

**DOI:** 10.1523/JNEUROSCI.2373-24.2025

**Published:** 2025-09-16

**Authors:** Melissa Henwood, Junkui Shang, Qiang Li, John Moth, John Henwood, Yang Yi, Dustin Green, Ajay Pal, Joseph Sandoval, Wei Li, Tiffany Dunn, Alfredo Sandoval, Jiewen Zhang, Subo Yuan, Bo Chen

**Affiliations:** ^1^Department of Neurobiology, University of Texas Medical Branch, Galveston, Texas 77555; ^2^Department of Neurosurgery, Massachusetts General Hospital, Harvard Medical School, Boston, Massachusetts 02114; ^3^Department of Anesthesiology, Houston Methodist Hospital, Houston, Texas 77030; ^4^Department of Neurology, Zhengzhou University People’s Hospital, Henan Provincial People’s Hospital, Zhengzhou 450001, China

**Keywords:** ascending projections, Brown–Séquard syndrome, sensory deficits, spinal cord injury

## Abstract

Spinal cord injury (SCI) often results in permanent sensory deficits, significantly impairing the quality of life. These deficits are poorly addressed due to a lack of valid animal models with translational relevance. Here, we utilized a thoracic Level 8 lateral hemisection SCI mouse model (including both male and female mice) and applied a battery of behavioral assays requiring supraspinal transmission of sensory information. We also assessed ascending spinal circuits from the lumbar spinal cord to the brain. By 28 d post-SCI, sensory assessments revealed distinct deficits: reduced innocuous sensation in the ipsilateral hindpaw and enhanced sensation in the contralateral hindpaw. Both hindlimbs exhibited disrupted nocifensive behaviors, with chronic neuropathic dysesthesia observed only in the contralateral hindlimb. We provided anatomical evidence to elucidate the neural substrates responsible for these sensory discrepancies. This SCI mouse model mimics key features of human lateral hemisection conditions (Brown–Séquard syndrome) and offers a robust platform to explore underlying mechanisms and develop new therapeutic strategies.

## Significance Statement

We present and validate a T8 lateral hemisection model that reproduces the hallmark sensory syndromes of Brown–Séquard syndrome (BSS). Systematic behavioral testing—spanning light-touch, nocifensive, and dysesthesia assays—combined with viral tracing of ascending pathways demonstrates that this single, reproducible lesion recreates the asymmetric sensory loss and chronic contralateral dysesthesia typical of BSS. By tightly matching clinical observations to preclinical readouts, the model offers a powerful platform for dissecting the mechanisms of SCI-induced sensory deficits and for evaluating targeted therapies.

## Introduction

Spinal cord injury (SCI) causes lasting motor and sensory impairments. While most preclinical work has focused on locomotor deficits (Donnelly and Popovich, 2008; Sofroniew, 2018; Courtine and Sofroniew, 2019; Fischer et al., 2020), sensory complications have been comparatively neglected. This gap is critical: SCI-related sensory problems—chronic dysesthesias (Stoè Rmer et al., 1997; Siddall and Loeser, 2001) and loss of light- and pleasant-touch perception that impairs sexual function (Anderson et al., 2007)—rank among patients’ top priorities (Lo et al., 2016). Moreover, enhanced sensory input can promote locomotor recovery (Takeoka and Arber, 2019; Takeoka, 2020). Progress, however, is hampered by a lack of animal models that faithfully replicate sensory deficits and allow mechanistic and therapeutic studies.

There are two key limitations in developing SCI models to study sensory deficits. First, unlike humans who can report sensory experiences, animal studies rely solely on quantifiable behavioral observations, which can be subjective and open to interpretation. Most sensory behavioral assays, such as hindpaw withdrawal tests, require functional hindlimb movement (Turner et al., 2019; Ma, 2022). However, SCI often results in hindlimb locomotor impairments, complicating the interpretation of behaviors and potentially leading to inaccurate conclusions. Second, although the sensory signals from various ascending tracts are well characterized within the spinal cords of rodent models (Todd, 2010; Abraira and Ginty, 2013; Chen et al., 2024), SCI typically results in highly variable lesions affecting different parts of the spinal cord (Schucht et al., 2002). This makes SCI unlikely to produce a predictable sensory deficit, especially using a clinically relevant model such as contusion. Thus, studying specific or multiple sensory experiences after SCI remains challenging. Collectively, these limitations highlight critical factors that should be considered in developing improved preclinical animal models, including (1) sufficient locomotor recovery for accurate interpretation of behavioral assays; (2) a consistent, predictable, and repeatable set of sensory readouts; and (3) the use of multiple approaches to validate the observed behavior.

In searching for a rigorous preclinical model of SCI-related sensory deficits, the thoracic lateral hemisection—which produces Brown–Séquard syndrome (BSS) in humans—stands out as an ideal candidate. In people with SCI, sensory sequelae such as loss of light touch, chronic dysesthesia, and even self-injury can be as debilitating as motor impairment. Lateral hemisection was first described more than two centuries ago for its dissociated sensory disturbances (Gowers, 1886) and, more recently, for its highly reproducible skilled locomotor-recovery trajectory (Little and Halar, 1985; Friedli et al., 2015). Because of that predictability, rodent hemisection has become a powerful platform for dissecting locomotor circuitry and testing therapeutic strategies (Ghosh et al., 2009; Filli et al., 2014; Takeoka et al., 2014; Liu et al., 2017; Squair et al., 2023). In contrast, far less is characterized about how this injury affects sensory modalities, such as light-touch perception, chronic dysesthesia, and sensory-related behavioral changes in animal models. Prior rodent studies (Christensen et al., 1996; Detloff et al., 2013; Martini et al., 2016; Brown et al., 2021; Lin et al., 2022) focused almost exclusively on hypersensitivity to mechanical or thermal stimuli and often report bilateral allodynia—findings at odds with the unilateral (contralateral-only) pain seen in BSS patients (Schott, 1993; Loch-Wilkinson et al., 2018). In addition, other work documented thermal stimuli-induced hindlimb hyposensitivity after lateral hemisectioned in mice (Vierck et al., 2013), further clouding the model’s clinical fidelity. Here we provide a comprehensive behavioral, functional, and anatomical analysis of light-touch perception, nocifensive responses, and neuropathic dysesthesia after T8 lateral hemisection in mice. Our goal is to establish a clinically faithful platform for probing the mechanisms of SCI-induced sensory loss and for developing targeted interventions.

## Materials and Methods

### Experimental method and subjects

All animal experiments and procedures were approved by the Institutional Animal Care and Use Committee of the University of Texas Medical Branch. All experimental procedures were performed in compliance with animal protocol approved by the Institutional Animal Care and Use Committee at University of Texas Medical Branch. Mice were housed in a temperature- and humidity-controlled animal facility with a 12 h light/dark cycle (6 AM lights on). Mice had *ad libitum* access to standard laboratory mouse diet pellets and water. Both male and female subjects were used, and littermates were randomly allocated to experimental groups. The 8–16-week-old adult mice were acquired from the following strains: wild-type C57BL/6 mice (Charles River Laboratories, strain code #000664). Mice were randomly assigned to experimental groups and observers were blinded where possible. Mice with over 15% weight loss after SCI were excluded from the study. Data were also collected and coded for subsequent blinded analysis. For our behavioral assessments, sample sizes were determined based on a priori power analysis (G*Power, University of Düsseldorf) to ensure a minimum power of 0.8. Furthermore, sample sizes were also determined based on prior physiological studies and our empirical experiences. For example, in the capsaicin behavior tests, which induce strong pain sensations and exhibit clear phenotypes, we reduced the sample size to *n* = 6 mice for animal welfare considerations. Conversely, for chronic neuropathic dysesthesia studies, we increased the power to 0.85 or higher, using *n* = 13 mice to provide robust evidence. All anatomical analyses were conducted with at least three to four biological replicates per experimental group, with specific *n* values listed in each figure legend.

### Surgical procedures

#### T8 lateral (right side) hemisection

Mice were anesthetized with ketamine/xylazine (100/10 mg/kg) and given a subcutaneous injection of 0.1 mg/kg buprenorphine SR for perioperative analgesia. A midline incision was made over the thoracic vertebrae, followed by a T8/9 laminectomy. For the T8 right-side hemisection, we carefully used both a scalpel and microscissors (Fine Science Tools) to interrupt the bilateral dorsal column (DC) at T8 and ensured no sparing of ventral pathways on the ipsilateral side. The muscle layers were then sutured, and the skin was secured with wound clips. All surgeries were completed by a researcher who was blind to the experimental conditions. After surgery, we maintained the animal’s body temperature using a second heating pad until fully recovered from anesthesia.

#### Intraspinal virus injection

AAV8-hSyn-mCherry-ChR2 and AAV-hSyn-EGFP-ChR2 (both at a titer of 1 × 10^12^ GC/ml) were obtained from the Boston Children's Hospital Viral Vector Core. At 6 weeks post-SCI, intraspinal viral injections were performed at the L3–L5 spinal cord levels. AAV8-hSyn-mCherry-ChR2 was injected into the left side, and AAV-hSyn-EGFP-ChR2 was injected into the right side of the same animal. Virus was injected using a UMP3 Ultra Micro Pump and Micro4 MicroSyringe Pump controller system (World Precision Instrument). This bilateral labeling approach allowed for direct comparison of viral expression across both sides of the spinal cord within each SCI mouse.

#### Postsurgical treatments and animal care

Prior to surgery, subjects were housed in same-sex groups of five with *ad libitum* access to chow and water. Upon the completion of surgical procedures, animal was administrated a subcutaneous injection of 1 ml of saline to mitigate dehydration risks. After surgery, mice were housed three mice per cage. Postoperative care involved diligent monitoring and interventions as required, ensuring the well-being of the animals. Mice with SCI had their bladders manually expressed twice daily until continence was regained (∼ 5 d) and moistened food was made available to encourage eating.

The subsequent postoperative observations were made with a focus on:

1. General activity levels of the animals
2. Incidences of aggressive interactions or fights among the animals
3. The condition of surgical wounds observing for signs of healing or complications
4. The presence of biting wounds
5. Signs of urinary infections or complications

In cases where open wounds or signs of infection were detected, a course of antibiotic therapy was initiated using Baytril at a dosage of 10 mg/kg for 7 d. Mice with visible injuries were housed individually to prevent further complications. Additionally, body weight was carefully monitored and recorded on a weekly basis for each mouse.

### Spontaneous behaviors

#### Grooming assessment

Subjects were placed in a small enclosure with a clear plexiglass floor. A camera and light source was positioned underneath the enclosure to provide a bottom-up view of the mice and their hindpaws. The mice were isolated in a temperature-controlled room with the camera running to capture spontaneous grooming behaviors for 20 min. Grooming was defined as the mouse using its forepaws or mouth to clean body regions, including the back, legs, hindlimbs, and toes. Video recordings were analyzed by blinded observers to quantify the total time spent grooming each body region.

#### Self-injury monitoring

Mice exhibiting signs of injury—ranging from skin redness to lacerations—were separated and housed individually. These SCI mice were monitored daily for the progression of injuries. If the injury site persisted or worsened over a 7 d period, the injury was presumed to be self-inflicted. The location and severity of each injury were documented and included in the analysis for this study.

#### Toenail injury assessment

Using an in-house scoring system, the degree of toenail loss and the presence of any injury (inflammation, swelling, or subungual bleeding) were recorded for each toe on both hindpaws following hemisection SCI. Prior to surgery, animals were acclimated to gentle hindpaw manipulation using surface restraint (tail hold). Any mouse exhibiting hindpaw nail breakage or visible injury before surgery was excluded from the study. Toenail assessments were conducted daily for 4 weeks during routine health checks and then 2–3 times per week thereafter. Each toenail was scored using the following scale: 0, normal appearance (curved and sharp); 1, blunted tip with preserved overall nail curvature; 2, rounded or flat tip with partial loss of nail curvature; 3, complete loss of nail curvature; 4, only a small nail stump remains; and 5, little or no visible nail. Up to two additional points were assigned per toe for the presence of inflammation/swelling (+1) and subungual bleeding (+1). For each hindpaw, a total score was calculated as the average of the five individual toe scores.

#### Dynamic weight bearing assessment

To assess animals for changes in paw weight support, we used the *Bioseb* Dynamic Weight Bearing system to assess hindpaw weight support and ground contact. Subjects were assessed prior to surgery (baseline) and the end of the 4 week study. A subset of hemisected animals were followed out to 12 weeks postinjury (*n* = 3). Mice were placed in a small chamber over a recording electrode for 5 min. The percentage of body weight support on each hindlimb and the area of paw contact with the floor electrode were recorded during this period.

### Motor assessment

Motor function was evaluated weekly with a locomotor open-ﬁeld rating scale, the Basso Mouse Scale (BMS). All BMS behavior tests were examined by investigators blinded to the treatment groups.

Whole-body kinematics: mice walking/running on treadmill were recorded using the high-speed motion capture system Vicon (Vicon Motion Systems), combining eight infrared cameras (200 Hz). The 3 mm reﬂective markers were attached bilaterally overlying the iliac crest: the greater trochanter (hip), the lateral condyle (knee), the malleolus (ankle), and the base of the metatarsal phalangeal joint (MTP) for the hindlimbs; the proximal head of the humerus (shoulder) and epicondyle of the humerus (elbow) and on the medial head of metacarpal (forepaw) for the forelimbs; and on top of the T12 vertebral bone (back). The 3D position of the markers was reconstructed ofﬂine using the Vicon Nexus software. The body was modeled as an interconnected chain of rigid segments. For subsequent kinematic analysis, parameters related to hindlimbs and the trunk were analyzed. Parameters (Fig. S1) describing gait timing, joint kinematics, and limb endpoint trajectory were computed for each gait cycle using custom-written MATLAB scripts, as reported previously (Brommer et al., 2021). Mice continuously walk on a treadmill and remained on the treadmill for >10 steps.

### Sensory assessment

#### Pinprick test

Each mouse was habituated in a small (7.5 × 7.5 × 15 cm) plastic cage for at least 20 min before testing. Sensitivity to noxious stimuli was tested at the end of the study period to prevent interference with other sensory assessments. Reactivity to pinprick stimulation and capsaicin injection were used to assess pain sensitivity. Hemisected subjects were compared with an intact cohort for analysis. Subjects were placed on a wire grid, and one hindpaw was probed with an insect pin (Austerlitz pin; size, 1). The percentage of paw withdrawal rate over five trials was reported.

Capsaicin sensitivity: subjects were briefly restrained, and a 0.025% capsaicin solution was intradermally injected into the plantar hindpaw. Paw licking was quantified for 30 min postinjection using video analysis. Mice were tested only once on each test, with different cohorts of mice used for each respective test.

#### Von Frey test

**Figure 1.**
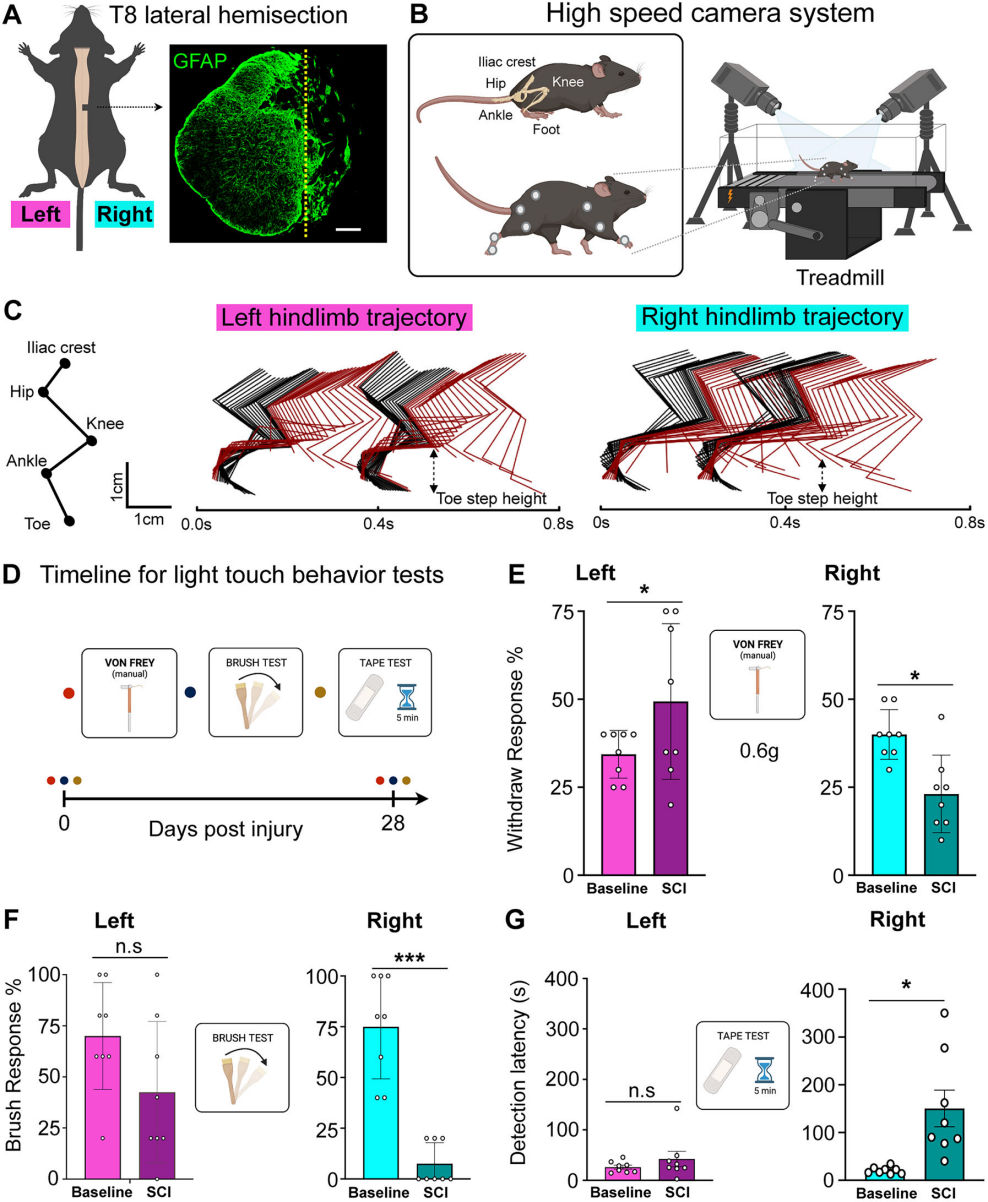
Impairment of innocuous sensitivity in the ipsilateral hindlimb following T8 lateral hemisection. ***A***, Left, A schematic illustrating the T8 lateral hemisection. Right, A representative transverse section at the lesion epicenter, stained with anti-GFAP. Scale bar, 100 µm. ***B***, Illustration of mice locomotion capture and recording setup using a high-speed camera system. ***C***, A representative color-coded stick figure decomposition showing both left and right hindlimb movements from a SCI mouse during two consecutive steps, 28 d post-SCI. ***D***, Schematic timeline for light-touch behavior tests. ***E***, Left, Post-SCI, mice show a significant increase in paw withdrawal response to a 0.6 g von Frey filament (*p* < 0.05) compared with baseline preinjury. Right, Post-SCI, mice show a significant decrease in paw withdrawal response to a 0.6 g von Frey filament. ***F***, Left, No significant changes in paw withdrawal response to dynamic brush pre- and post-SCI. Right, A significant decrease in paw withdrawal response to dynamic brush observed pre- and post-SCI. ***G***, Left, No significant changes in detection of tape on the left paw before and after SCI. Right, Post-SCI, mice show a significant increase in time taken to detect tape on the right paw. For (***E–G***) *n* = 8 mice per group, each dot represent a mouse, two-tailed paired *t* test for all tests. Error bars indicate standard deviation (SD).

Each mouse was habituated in a small (7.5× 7.5 × 15 cm) plastic cage for at least 20 min before testing. For Figure 1*E*, to screen for dysesthesia in each hindpaw, we applied a single 0.6 g von Frey filament applied to both SCI mice hindlimbs and calculated the percentage of paw withdrawals. Each filament was tested 10 times. Between individual measurements, von Frey filaments were applied at least 3 s after the mice had returned to their initial resting state. A 0.6 g force is widely regarded as a “watershed” stimulus (Boada and Woodbury, 2008; Liu et al., 2018)—the boundary between light-touch and noxious input. Thus, this single-force assay provides a rapid screen for SCI-induced alterations in light-touch sensitivity. For Figure 3*B*, the von Frey test used a set of nylon ﬁlaments (0.008, 0.02, 0.04, 0.07, 0.16, 0.4, 0.6, 1.0, 1.4 g) to stimulate the hindpaw plantar surface of each mouse in order to determine the 50% withdrawal threshold. Mice were tested with a series of ﬁlaments using the “up–down” method of Dixon, as described previously (Mogil et al., 1999). This continuous measure provides the intensity of mechanical hypersensitivity (lower threshold) or hyposensitivity (higher threshold) in each paw. Investigators were blinded to the treatment groups.

#### Brush test

Each mouse was habituated in a small (7.5 × 7.5 × 15 cm) plastic cage for at least 20 min before testing. The plantar hindpaw was stimulated by light stroking from heel to toe with a number 4 round paintbrush. For baseline dynamic mechanical sensitivity, the test was repeated 10 times. The number of occurrences of walking away or occasionally brief paw lifting was measured.

#### Hargreaves test

The radiant heat paw withdrawal test involved stimulating the hindpaw plantar surface with a focused beam of light (set to 30% maximum intensity of the machine) and verifying latency to withdraw either hindpaw. Each hindpaw was stimulated three times, and data were represented as the average of three withdrawal latencies, with a three intertrial interval of at least 2 min, performed as previously described (Hargreaves et al., 1988). Observers were blinded to treatment paradigm.

#### The adhesive removal test

Mice were tested on 5 consecutive days for the ability/motivation to remove an adhesive placed on the plantar surface of the hindpaw. The adhesive was half of a 1.5 ml Eppendorf tube cap label, cut into a semicircle, and placed on the plantar surface. The latency to remove the label was recorded to the nearest minute, and these data were reported exactly as recorded (with only one test per day and no averaging between trials). If mice did not remove the adhesive within 30 min of the start of the test, latency was recorded as “30 min” for the purpose of data analysis, and mice were then returned to their home cage.

#### The hot plate test

Mice were placed within the hot–cold plate apparatus (Bioseb) on a stainless-steel metal plate heated to 53 ± 0.1C or cooled to 0 ± 0.1C and were video-recorded from the side (with a mirror opposite the test chamber to view each side of the mouse) for 60 s at which point they were returned to their home cage. The latency to either lick the hindpaw, ﬂutter of the hindpaw, or to attempt to escape via jumping was recorded. Mice were tested once, and data were represented directly based on behaviors recorded in one 60 s trial. Entirely different cohorts of mice were used for the hot plate tests, respectively, to prevent behavioral adaptation to the test.

#### The dry ice test

Briefly, dry ice vapor was directed toward the tested hindpaw from a distance of 0.3–0.5 cm without making contact. The latency between the application of the vapor and the withdrawal of the hindpaw was recorded.

### Immunohistochemistry

For immunohistochemistry studies, mice were perfused at the end of experiments. Mice were deeply anesthetized by an intraperitoneal injection of ketamine/xylazine (100/10 mg/kg). Mice were transcardially perfused with PBS followed by 4% paraformaldehyde (PFA) in PBS. The dissected spinal cords and brains were postfixed in 4% PFA overnight, followed by immersion in 30% sucrose solution for at least 2 d before embedding in plastic molds (Thermo Fisher Scientific number 22-19). For coronal sections, spinal cords were cut into continuous and mounted with the rostral side facing the bottom. All tissues were snap-frozen in a bathtub of dry ice-dehydrated ethanol. The tissues were then continuously cryosectioned (Leica, CM1950) into 40 µm sections for floating staining. To prepare the tissue for staining, it was blocked for 1 h in PBS containing 10% donkey serum and 0.5% Triton X-100. Subsequently, the tissue was incubated overnight at 4°C in primary antibodies and diluted in PBS with 10% donkey serum and 0.5% Triton X-100. After three 5 min washes, the tissue was treated with secondary antibodies for 2 h at room temperature. The primary antibodies used were as follows: rat anti-GFAP [Invitrogen (13-0300), 1:600]; chicken anti-GFP [Abcam (ab13970), 1:400]; and rabbit anti-RFP [Abcam (ab34771), 1:400]. The secondary antibodies used were 647 donkey anti-guinea pig (Jackson ImmunoResearch Laboratories number706-605-148), 555 donkey anti-rabbit (Invitrogen number A-31572), and 488 donkey anti-rat (Invitrogen number A-21208). After staining, the slides or floating tissues were washed. They were then adhered to room-temperature charged microscope slides and mounted with Fluoromount-G Mounting Medium (Invitrogen number 00-4959-52). Fluorescent images were acquired using an inverted microscope (Nikon Eclipse Ti2) or a confocal laser-scanning microscope (LSM880, Carl Zeiss) in the Optical Microscopy Core of UTMB. The brightness and contrast of the images were adjusted, pseudocolors were applied for presentation purposes, and the images were cropped using ImageJ (NIH). During image quantification, image capture and processing parameters were kept constant. For each spinal cord, 4–5 slides were imaged across the dorsal–ventral axis.

## Results

### Thoracic lateral hemisection mouse model showed spontaneous locomotor recovery

BSS (Brown-Séquard, 1868) is often caused by unilateral spinal cord lesions that result in predictable asymmetrical sensory deficits and significant motor recovery, making it an ideal condition to model. To model BSS, we performed a lateral hemisection injury at the thoracic Level 8 because the resultant lesions and locomotor deficits are reproducible and quantifiable (Bareyre et al., 2004; Vierck et al., 2013; Brown and Martinez, 2018; Cao et al., 2021). Despite some variation between subjects, lesions were primarily confined to the right side (Fig. 1*A*).

We used this lateral hemisection model to monitor locomotor recovery (Fig. S1*A*). From Day 0 to 7 postinjury, mice initially showed paralysis (BMS 0) but gradually improved, exhibiting dorsal stepping (BMS 3). From 7 to 28 d post-SCI, mice progressed from dorsal stepping to plantar stepping (BMS 5) and coordinated walking with hindlimbs (BMS 6-8). By 28 d post-SCI, SCI mice (100%) returned to near baseline levels, indicating nearly complete recovery (repeated-measure one–way ANOVA, *F*_(1.693, 8.465)_ = 112.8; *p* < 0.0001; Bonferroni’s post hoc, BL vs 7 d, *t*_(5)_ = 16.81; *p* < 0.0001; BL vs 14 d, *t*_(5)_ = 13.56; *p* = 0.0002; BL vs 21 d, *t*_(5)_ = 7.319; *p* = 0.0030; BL vs 28 d, *t*_(5)_ = 1.581; *p* = 0.1696; Fig. S1*B*). These results are consistent with the degree of locomotor recovery seen in BSS patients, who initially suffer from complete paralysis but gradually recover, with 75–90% achieving unaided walking in the chronic stage post-SCI (Little and Halar, 1985). Voluntary paw withdrawal is required for rodent sensory testing (Nakae et al., 2011; Shiao and Lee-Kubli, 2018; Turner et al., 2019; Ma, 2022), so we assessed hindlimb kinematics at 28 d post-SCI (Fig. 1*B*). High-speed video and 3D reconstruction showed clear “foot-off-ground” swings in both legs (Fig. 1*C*; Movie 1), although ipsilateral step height was modestly reduced relative to the baseline. In the contralateral (left) leg, paired *t* tests showed the following: Mouse 1, *t*_(22)_ = 2.257; *p* = 0.0343; Mouse 2, *t*_(14)_ = 1.265; *p* = 0.2267; Mouse 3, *t*_(10)_ = 0.560; *p* = 0.5881; Mouse 4, *t*_(16)_ = 7.791; *p* < 0.000001; Mouse 5, *t*_(18)_ = 2.229; *p* = 0.0388; and Mouse 6, *t*_(15)_ = 0.237; *p* = 0.8158. In the ipsilateral (right) leg, paired *t* tests showed the following: Mouse 1, *t*_(22)_ = 10.30; *p* < 0.000001; Mouse 2, *t*_(15)_ =3.336; *p* = 0.00452; Mouse 3, *t*_(8)_ = 12.38; *p* = 0.000002; Mouse 4, *t*_(17)_ = 4.782; *p* = 0.000173; Mouse 5, *t*_(19)_ = 6.965; *p* = 0.000001; and Mouse 6, *t*_(15)_ = 2.852; *p* = 0.01211 (Fig. S1*C*,*D*). Overall gait cycle timing between hindlimbs was unchanged (paired *t* test, *t*_(5)_ = 0.441; *p* = 0.6616; Fig. S1*E*; *t*_(5)_ = 0.4648; *p* = 0.4987; Fig. S1*F*), with only a shorter swing phase in the ipsilateral leg (paired *t* test, *t*_(5)_ = 1.524; *p* = 0.1880; Fig. S1*G*; *t*_(5)_ = 5.217; *p* = 0.0034; Fig. S1*H*)—likely due to diminished toe lift. Thus, despite mild asymmetry, hemisectioned mice retain adequate voluntary hindlimb elevation, validating their use for chronic-phase sensory assays.

**Movie 1.** Representative behaviors in mice with lateral hemisection SCI. This video includes three clips showcasing key behavioral phenotypes: (1) overground walking, (2) responses on the hot plate test, and (3) spontaneous grooming behavior. These clips illustrate motor and sensory changes following thoracic lateral hemisection. [View online]

### Lateral hemisection selectively impairs light-touch sensitivity in the ipsilateral hindlimb

Sensory deficits in patients with BSS include reduction of light touch and proprioception sensation ipsilateral to the injury (Ahuja et al., 2017). To evaluate changes in innocuous sensitivity in hemisected mice, we conducted a series of sensory tests on both the ipsilateral and contralateral hindlimbs of mice 28 d after SCI. Prior to surgery, the same cohort of mice underwent identical behavioral tests prior to the SCI to establish a baseline (Fig. 1*D*). The unilateral SCI resulted in a specific and marked reduction in behavioral responses to both dynamic (paired *t* test, *t*_(7)_ = 3.406; *p* < 0.0001) and static (paired *t* test, *t*_(7)_ = 3.320; *p* = 0.0128) light touch in the ipsilateral hindpaw. Conversely, the contralateral hindpaw preserved its withdrawal response to dynamic (paired *t* test, *t*_(7)_ = 1.823; *p* = 0.1112) and static (paired *t* test, *t*_(7)_ = 2.510; *p* = 0.0404; post-SCI was more sensitive) light touch when compared with before SCI (Fig. 1*E*,*F*).

To independently verify the loss of tactile perception in the ipsilateral hindpaw of SCI mice, we conducted a tape removal task, measuring the time it takes for mice to detect and attempt to remove the tape stuck to the dorsal surface of their hindpaw (Liu et al., 2018). Before SCI, mice typically recognized and tried to remove the tape within ∼20 s. However, following a T8 lateral hemisection, the latency significantly increased; mice took an average of 165 s to detect the tape on the ipsilateral hindpaw (paired *t* test, *t*_(7)_ = 3.403; *p* = 0.0114). This marked decline in tactile sensitivity was contrasted by the prompt response (∼20 s) to tape on the contralateral hindpaw (paired *t* test, *t*_(7)_ = 0.9176; *p* = 0.3893), highlighting the sensory deficits specifically associated with the injured side (Fig. 1*G*). In summary, our results indicate that unilateral T8 spinal cord hemisection injuries in mice lead to a significant and selective impairment of light-touch sensory responses in the ipsilateral hindpaw, while the contralateral hindpaw remains unaffected.

### Lateral hemisection impairs nocifensive behaviors in both hindlimbs

**Figure 2.**
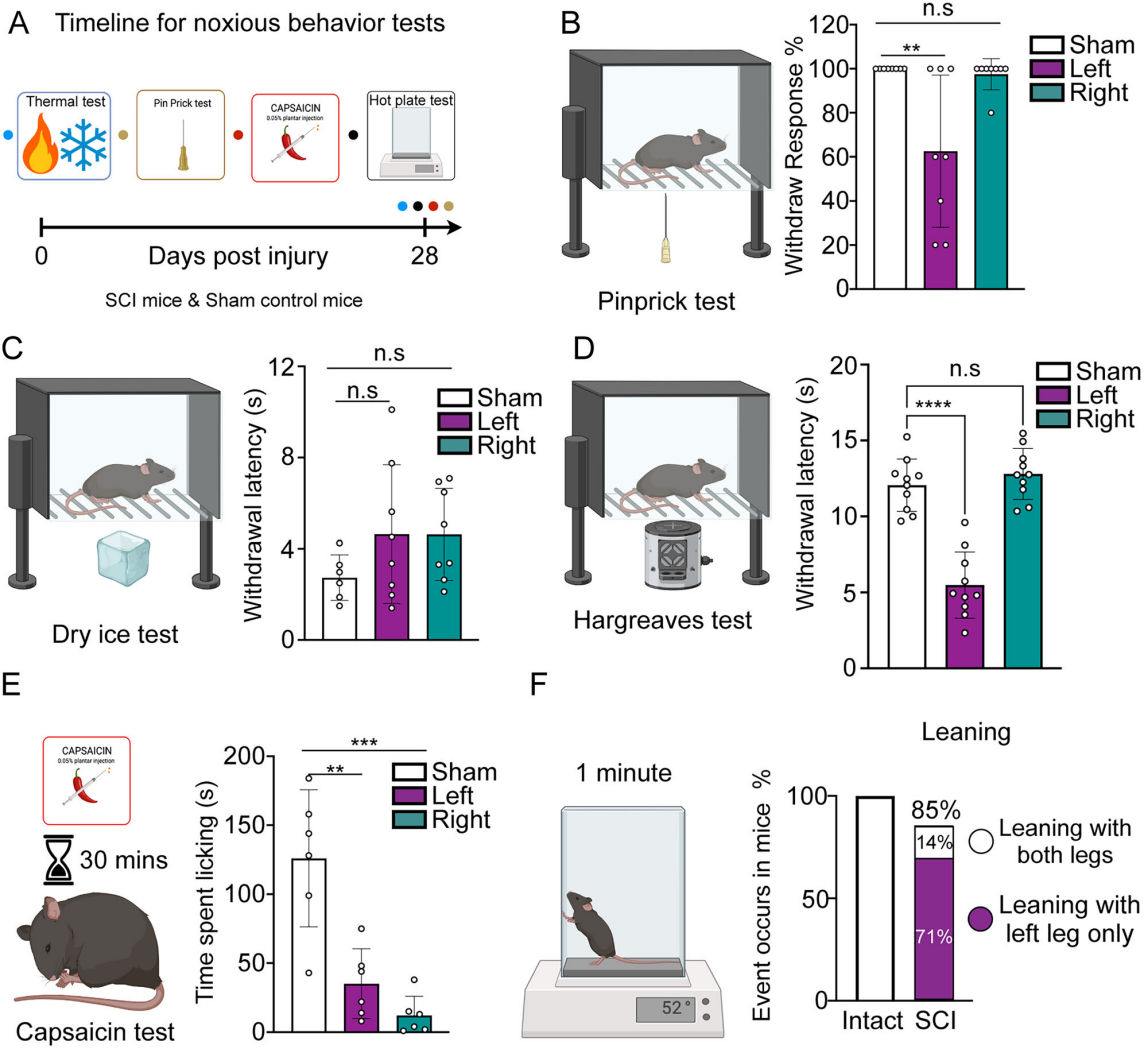
Impairment of nocifensive behaviors in hindlimbs following T8 lateral hemisection. ***A***, Schematic timeline detailing the schedule for nocifensive behavior tests. ***B–F***, Left, Schematic for behavior tests. Right, Quantitative results of the specific behaviors. ***B***, Displays a significant reduction in response to pinprick in the left hindpaw, with no significant change in the ipsilateral hindpaw. ***C,D***, No detectable changes in response to dry ice (***C***) and Hargreaves (***D***) tests. ***E***, Significantly reduced licking responses to capsaicin injection in both hindpaws over 30 min. ***F***, Defensive behaviors, leaning and jumping responses during a 1 min hot plate test remained intact in SCI mice. For panels ***B–D***, *n* = 8 mice per group, with each dot representing an individual mouse. Statistical analysis was performed using one-way ANOVA followed by Bonferroni’s correction. Error bars indicate SD. For panel ***E***, *n* = 6 mice per group, with each dot representing an individual mouse, one-way ANOVA, and Bonferroni’s correction. Error bars indicate SD. For panel ***F***, *n* = 7 mice per group.

In BSS, unilateral spinal injury abolishes pinprick and temperature sensation contralaterally. To probe comparable changes in our T8 hemisection mice, we first tested pinprick responses 28 d postinjury. Withdrawal from a noxious needle fell to an average of 63% in the contralateral paw versus 100% in shams, whereas ipsilateral withdrawal remained near normal (98%; ne-way ANOVA, *F*_(2,21)_ = 8.489; *p* = 0.0020; Bonferroni’s post hoc, sham vs contralateral; *p* = 0.0026; sham vs ipsilateral; *p* = 0.9569; Fig. 2*A*,*B*). We then assessed thermal nociception. Standard cold stimulation (dry ice evaporation) revealed no differences (one-way ANOVA, *F*_(2,19)_ = 1.526; *p* = 0.2428; Dunnett’s post hoc, sham vs contralateral, mean diff. = −1.908; *p* = 0.2263; sham vs ipsilateral, mean diff. = −1.894; *p* = 0.2304; Fig. 2*C*), but the Hargreaves assay showed contralateral heat hyperalgesia with no ipsilateral change (one-way ANOVA, *F*_(2,27)_ = 46.00; *p* < 0.0001; Bonferroni’s post hoc, sham vs left, *p* < 0.0001; sham vs right, *p* = 0.5885; Fig. 2*D*). Because withdrawal reflexes can be spinally mediated—even after complete transection (Barik et al., 2018)—we next injected the TRPV1 agonist capsaicin into a single hindpaw. Sham mice licked the injected paw for >120 s; SCI mice licked markedly less (left, <40 s; right, <20 s), indicating bilateral impairment of nocifensive coping (one-way ANOVA, *F*_(2,15)_ = 19.77; *p* < 0.0001; Bonferroni’s post hoc, sham vs contralateral, *p* = 0.0005; sham vs ipsilateral, *p* < 0.0001; Fig. 2*E*). Finally, we used a 52°C hot plate, which requires supraspinal processing (Espejo and Mir, 1993; Langford and Mogil, 2008; Casarrubea et al., 2019). Hindpaw licking fell from 100% (sham) to 28.6% (SCI), but exploratory, forepaw licking, leaning, and jumping were preserved (Fig. S2). Strikingly, 71% of SCI mice leaned exclusively on the contralateral paw (Fig. 2*F*; Movie 1), showing greater heat tolerance in that limb. Thus, despite assay-specific differences, T8 hemisection produces an asymmetric sensory profile: contralateral loss of mechanical nociception and contralateral heat hyperalgesia by Hargreaves yet overall bilateral reduction in sustained nocifensive behaviors, consistent with the mixed-sensory deficits observed clinically in BSS.

### Lateral hemisection induces chronic dysesthesia in both hindlimbs

**Figure 3.**
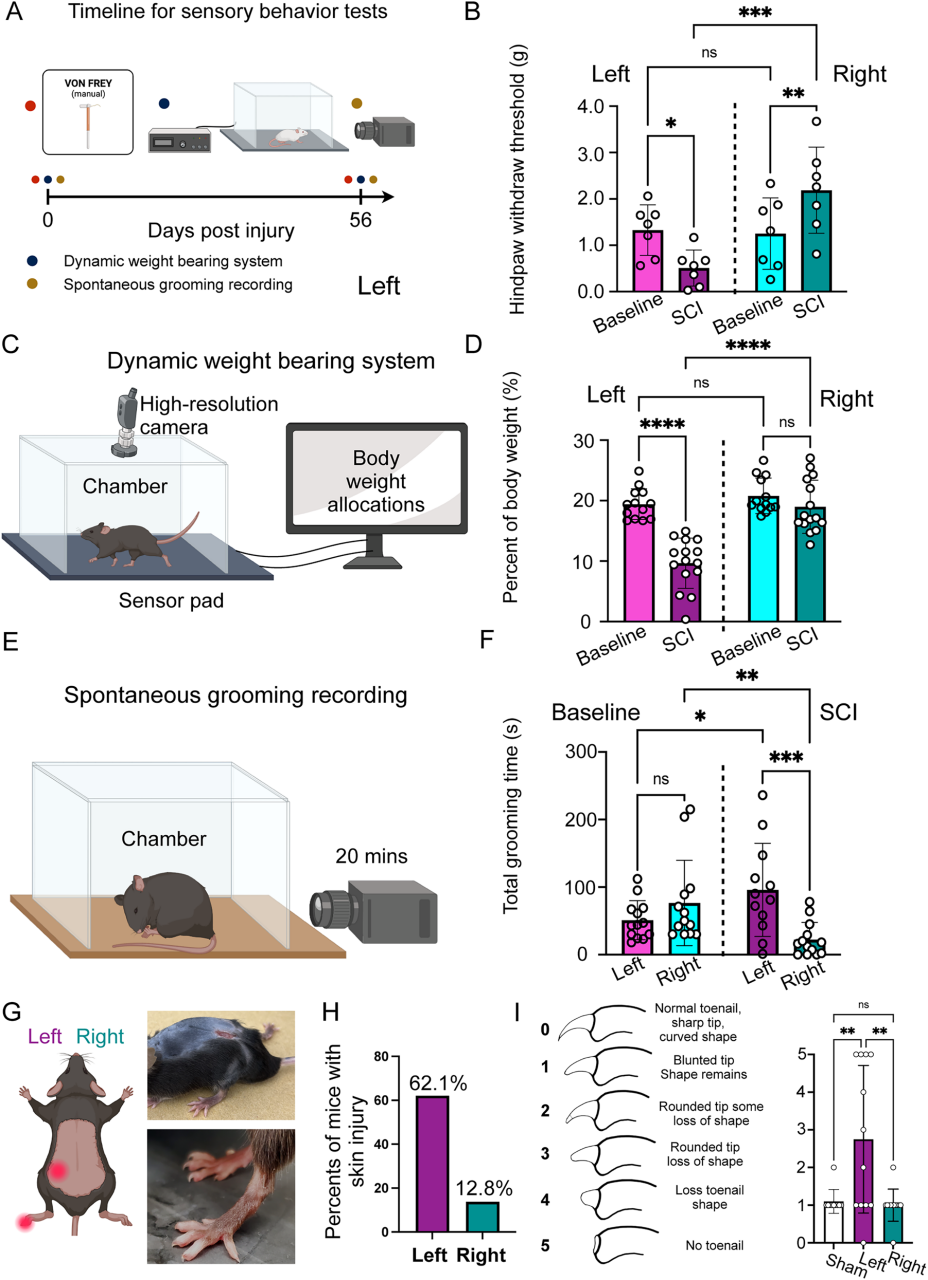
Induction of chronic dysesthesia in hindlimbs following T8 lateral hemisection. ***A***, Schematic timeline of the chronic dysesthesia testing schedule. ***B***, Paw withdrawal thresholds (PWTs) to von Frey filaments: the left paw shows a significant decrease, whereas the right paw shows an increase (*n* = 7/group). ***C***, Schematic of the dynamic body-weight-support assay. ***D***, Body weight allocation is significantly reduced on the left paw, with no change on the right paw (*n* = 13/group). ***E***, Schematic of the setup for monitoring spontaneous grooming. ***F***, Grooming becomes asymmetric after SCI, with a preference for the left side of the body (*n* = 13/group). ***G***, Quantification of self-injury sites in SCI mice, left versus right (*n* = 13). ***H***, Representative images of chronic injury sites. ***I***, Toenail-biting score (left) and quantification, showing severe biting of left toenails after SCI (*n* = 13). Statistical analysis. For panels ***B***, ***D***, and ***F***, each dot represents one mouse; two-way repeated–measure ANOVA with Bonferroni’s correction. ***I***, One-way ANOVA with Bonferroni’s correction. Error bars indicate SD.

A recent clinical study developed a new colored pain drawing tool to differentiate neuropathic dysesthesia among various types of patients, including those with BSS. In the report, the authors highlighted that BSS patients frequently experience persistent burning, stabbing, and tingling sensations in the contralateral leg, from hip to toes, while experiencing numbness in the ipsilateral leg (Sehgal et al., 2021). To investigate this phenomenon in a lateral hemisection mouse model, we conducted von Frey testing at 8 weeks post-SCI. A two-way repeated–measure ANOVA revealed a significant interaction between side and condition (*F*_(1,12)_ = 18.14; *p* = 0.0011) and a main effect of side (*F*_(1,12)_ = 6.861; *p* = 0.0224), but no main effect of condition (*F*_(1,12)_ = 0.0855; *p* = 0.7749). Post hoc Fisher's LSD tests showed decreased mechanical thresholds in the ipsilateral hindpaw from the baseline to SCI (*t*_(12)_ = 3.219; *p* = 0.0074) and increased thresholds in the contralateral hindpaw (*t*_(12)_ = 2.805; *p* = 0.0159). Thresholds did not differ between sides at the baseline (*t*_(24)_ = 0.205; *p* = 0.8390), but after SCI, ipsilateral thresholds were significantly lower than contralateral thresholds (*t*_(24)_ = 4.551; *p* = 0.0001; Fig. 3*A*,*B*). This heightened sensitivity in the contralateral leg may be attributed to chronic neuropathic dysesthesia or overuse of the contralateral unaffected leg, a common issue among SCI patients (Watson and Sandroni, 2016). To distinguish between these possibilities, we used a dynamic weight-bearing system (Fig. 3*C*) to assess body weight distribution during free walking before and after SCI. A mixed-effect model revealed significant main effects of limb side (row factor, *F*_(1,28)_ = 25.32; *p* < 0.0001) and injury (column factor, *F*_(1,24)_ = 43.52; *p* < 0.0001), as well as a significant interaction (*F*_(1,24)_ = 20.23; *p* = 0.0001). Post hoc comparisons showed that contralateral weight-bearing decreased markedly after SCI (baseline vs SCI, mean difference = 9.864 ± 1.257; *t*_(24)_ = 7.845; *p* < 0.0001), whereas the ipsilateral limb showed no significant change (baseline vs SCI, mean difference = 1.866 ± 1.257; *t*_(24)_ = 1.484; *p* = 0.1507). At the baseline, left and right hindlimb loads were similar (*p* = 0.3547), but after SCI, contralateral load was significantly lower than ipsilateral (mean difference = −9.333 ± 1.335; *t*_(52)_ = 6.991; *p* < 0.0001; Fig. 3*D*). Thus, SCI mice shift weight away from the contralateral hindlimb without increasing load on the ipsilateral side, arguing against compensatory overuse. Post-SCI mice exhibited a marked lateralized imbalance in grooming, with excessive plucking and biting directed toward the left side of the body, in contrast to the symmetrical grooming seen pre-SCI. Mixed-effect analysis revealed a significant injury × side interaction (*F­*_(_₁,₂₄_)_ = 20.23; *p* = 0.0004), with no main effect of injury (*F*_(_₁,₂₈_)_ = 0.10; *p* = 0.7588) or side (*F*_(_₁,₂₄_)_ = 4.02; *p* = 0.0577). Post hoc Fisher's LSD tests confirmed greater left–right asymmetry in SCI mice (*t*_(_₂₃_)_ = 4.297; *p* = 0.0003) but not in baseline controls (*t*_(_₂₃_)_ = 1.548; *p* = 0.1353) and significant side-specific changes in grooming durations for both the left (*t*_(_₄₇_)_ = 2.220; *p* = 0.0313) and right (*t*_(_₄₇_)_ = 2.765; *p* = 0.0081) sides relative to the baseline (Fig. 3*F*; Movie 1). These spontaneous aggressive behaviors resulted in multiple skin injuries on the contralateral body side (Fig. 3*G*), especially in the hip and toenails (Fig. 3*H*,*I*), indicating that SCI may induce self-injury behaviors. Quantification of toenail conditions confirmed significant differences among groups (*F*_(_₂,₃₁_)_ = 7.764; *p* = 0.0018; *R*² = 0.3337), with the contralateral hindpaw showing broken nails than both shams (*t*_(_₃₁_)_ = 3.193; *p* = 0.0097) and ipsilateral hindpaws (*t*_(_₃₁_)_ = 3.552; *p* = 0.0037), while the ipsilateral toenail did not differ from shams (*t*_(_₃₁_)_ = 0.194; *p* > 0.9999). Remarkably, the pattern of these injuries closely mirrors the dysesthesia distribution reported by human BSS patients (Sehgal et al., 2021). This correlation suggests that the lateral hemisection mouse model effectively replicates the chronic neuropathic dysesthesias observed in human BSS.

### Lateral hemisection disrupts ipsilateral spinal mono-synaptic ascending projections

**Figure 4.**
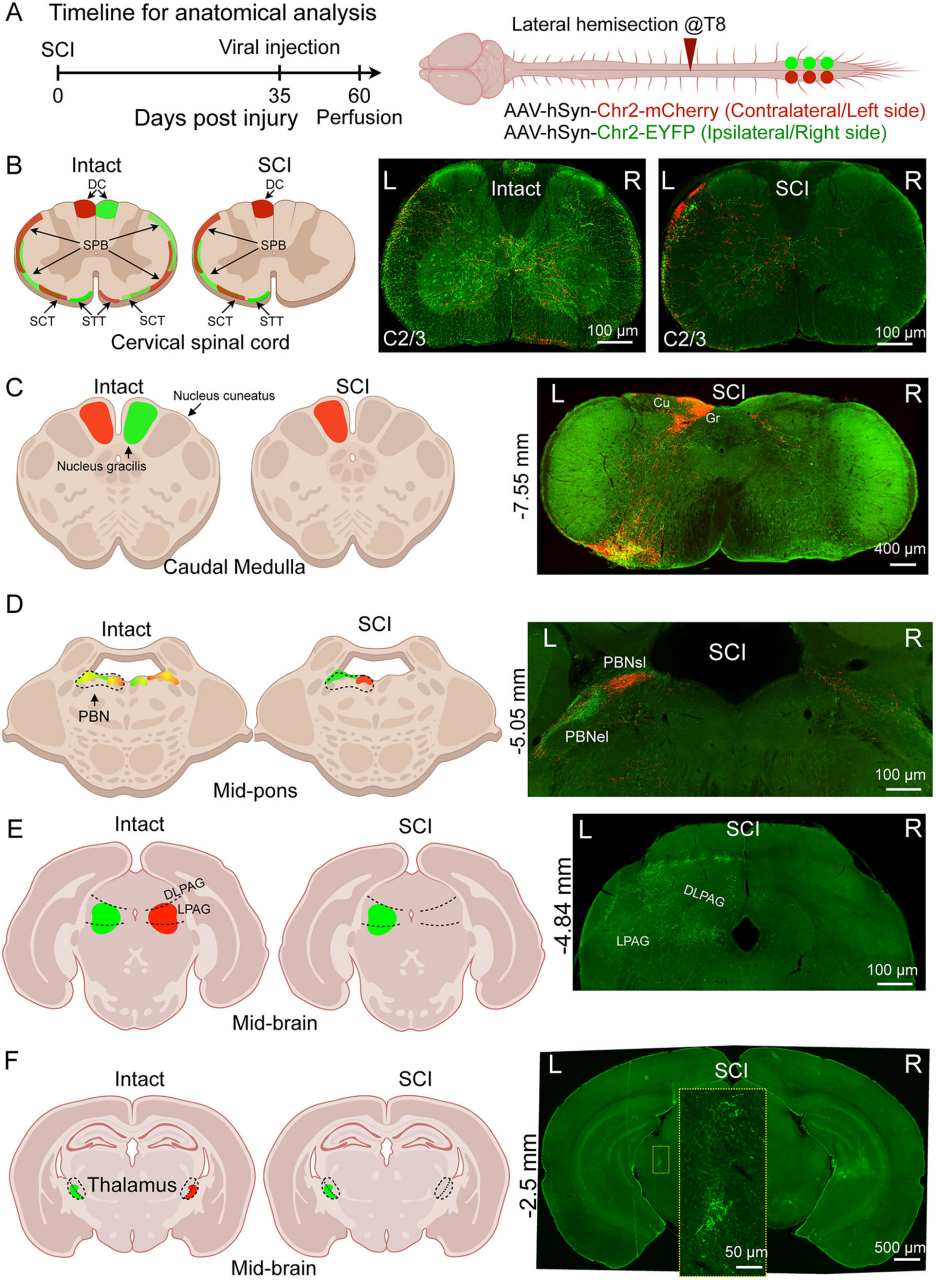
Disrupted ipsilateral spinal mono-synaptic ascending projections in SCI mice. ***A***, Left, Schematic timeline of AAV-based tracing experiments in SCI and sham control mice. Right, Diagram of AAV injection strategy to anterogradely label spinal projection neurons. Green represents ipsilesional projections; red represents contralesional projections. ***B***, Left, Schematic of ascending spinal tracts in the cervical spinal cords. Right, Representative transverse section of cervical spinal cords stained for GFP (green) and RFP (red), highlighting the distribution of labeled axons. Scale bar, 100 µm. ***C–F***, Left, Diagrams showing the termination of ascending spinal tracts at different coronal brain sections with distances from the bregma indicated. Right, Representative brain sections stained for GFP and RFP at various bregma levels, −7.55 mm (***C***), −5.05 mm (***D***), −4.84 mm (***E***), and −2.5 mm (***F***). Scale bars are provided in the figures. *n* = 3 (intact) and 4 (SCI) mice.

To validate our interpretations of SCI mouse behaviors, we leveraged the well-characterized anatomical basis for sensory and emotional experiences (Abraira and Ginty, 2013; Barik et al., 2018; Koch et al., 2018; Choi et al., 2020; Chen et al., 2024). To label spinal projection neurons that respond to noxious and innocuous stimuli, we injected an AAV encoding mCherry into the right side of the spinal cord and an AAV encoding EGFP into the left side at the lumbar enlargement in both SCI and intact mice (Fig. 4*A*; Fig. S3*A*). This allowed us to visualize several major ascending tracts in both intact mice and 8 weeks post-SCI, as shown in images of the cervical spinal cords (Fig. 4*B*). In intact mice, we observed major ascending tracts from both sides, including the ipsilateral postsynaptic DC pathway, the ipsilateral spinocerebellar (SCT) pathway, the bilateral spinoparabrachial (SPB) pathway, and the contralateral spinothalamic (STT) pathway. In sharp contrast, SCI mice showed the left ipsilateral DC, SCT, and SPB pathways, as well as the right side STT and SPB (contralateral projection) pathway, while the left STT pathway and right DC, SCT, and SPB (ipsilateral projection) pathways were absent (Fig. 4*B*).

We next traced axons of lumbar spinal projection neurons to brain regions responsible for light touch (Abraira and Ginty, 2013; Paixão et al., 2019), noxious stimuli (Todd, 2010; Roome et al., 2020), nocifensive coping (Barik et al., 2018; Deng et al., 2020; Roome et al., 2020), and defensive (Tovote et al., 2016) behaviors. The DC nuclei in the brainstem receive ipsilateral spinal projections and are responsible for light touch and tactile stimulation (Abraira and Ginty, 2013; Paixão et al., 2019). In intact mice, mCherry and EGFP-labeled fibers were confined to their respective ipsilateral gracile nuclei (Fig. S3*B*). In contrast, SCI mice showed mCherry + fibers only in the left gracile nucleus, with a marked reduction of EGFP + fibers on the right side (Fig. 4*C*). These observations consistent with the results that the ipsilateral (right) side lost light-touch sensation, while the contralateral (left) side preserved it (Fig. 1*E*–*G*).

The parabrachial nucleus (PBN) directly receives bilateral spinal nociceptive signals, with the ipsilateral SPB pathway being critical for nocifensive coping behavior (Barik et al., 2018; Deng et al., 2020). In intact mice, mCherry + and EGFP + fibers overlapped in both left and right PBN. Notably, the distribution of ipsilateral projection fibers was more concentrated in the superior lateral PBN, while contralateral projections were more external lateral (Fig. S3*C*). In SCI mice, this distribution pattern was more pronounced on the left side of the PBN, with fluorescent fibers largely reduced on the right side (Fig. 4*D*). In the thalamus, which is responsible for discriminative pain (Willis et al., 1979; Willis and Westlund, 1997; Todd, 2010), intact mice showed fibers mostly on the contralateral side within the dorsal part of the ventral posterolateral (VPL) nucleus (Fig. S3*E*). SCI completely abolished mCherry + fibers in the VPL nucleus (Fig. 4*F*). Together, these results suggested that although both hindlimbs showed reduced coping behaviors after SCI, it is likely due to different spinal projection circuit mechanisms. The ipsilateral side coping behaviors (Fig. 2*B*,*E*) absence was due to disruption of the ipsilateral SPB pathway, while the contralateral side (Fig. 2*F*) might be due to the loss of discriminative pain.

Lastly, defensive behaviors, such as escaping in hot plate test, are believed to be controlled by periaqueductal gray (PAG) neurons (Tovote et al., 2016). In intact mice, fibers were primarily located in the contralateral lateral PAG and were present on both sides (Fig. S3*D*). In contrast, SCI disrupted only mCherry + fibers originating from the left side in the PAG (Fig. 4*E*) while preserving signals from the ipsilateral side. These anatomical observations may offer an explanation for why the defensive behaviors were preserved in the SCI mice (Fig. 2).

## Discussion

SCI-induced sensory deficits, such as chronic spontaneous dysesthesia/neuropathic pain and loss of sexual functions, are among the top functional priorities for patients (Lo et al., 2016). However, current SCI research and treatments primarily focus on improving locomotor recovery by enhancing brain descending input (He and Jin, 2016; Mahar and Cavalli, 2018; Sofroniew, 2018; Courtine and Sofroniew, 2019). Although several pioneering rodent models have explored post-SCI neuropathic pain (Christensen et al., 1996; Detloff et al., 2013; Martini et al., 2016; Lin et al., 2022), none capture the full spectrum of sensory disturbances, leaving this therapeutic domain underexplored. To bridge this gap, we sought to develop, characterize, and validate a mouse model that reproduces the sensory dysfunctions observed clinically. Leveraging the classical BSS—often cited in textbooks to illustrate how ascending tracts convey pain, temperature, and light touch—we performed a right-sided thoracic (T8) lateral hemisection. We comprehensively characterized sensory changes in mice subjected to a right-sided thoracic (T8) lateral hemisection and found that the lesion produces asymmetric sensory deficits in hindpaws—diminished light-touch detection on the ipsilateral side and impaired nocifensive responses on the contralateral side. Moreover, most injured mice developed chronic hindlimb dysesthesias, with >60% displayed self-injury directed exclusively at the nonlesioned limb. Finally, viral tracing of ascending pathways showed that these behavioral phenotypes map precisely onto the disrupted tracts, underscoring the model’s mechanistic fidelity. Collectively, our integrated behavioral and anatomical data reveal striking parallels between BSS patients and T8-hemisected mice (Table 1), establishing this model as a robust translational platform for mechanistic studies of SCI-induced sensory pathology and for preclinical evaluation of targeted therapies.

**Table 1.** Sensory deficits reported in patients with BSS

Legs	Functions			
	Motor functions	Light touch	Pain and temperature	Chronic dysesthesia
Ipsilateral side	Muscle weakness (Park et al., 2015; Nelson et al., 2020; Hoebee et al., 2022)	Reduced (Park et al., 2015; Nelson et al., 2020; Hoebee et al., 2022)	Normal (Park et al., 2015; Nelson et al., 2020; Hoebee et al., 2022)	Numbness (Sehgal et al., 2021)
Contralateral side	Normal (Park et al., 2015; Nelson et al., 2020)	Normal/allodynia (Schott, 1993)	Reduced (Park et al., 2015; Nelson et al., 2020; Hoebee et al., 2022)	Stabbing and tingling/pin and needles (Sehgal et al., 2021)

T8 lateral hemisection is a valuable tool for studying the underlying mechanisms of SCI-induced sensory deficits. The rodent contusion (or lateral contusion) SCI model is commonly used to study underlying mechanisms of SCI-induced neuropathic pain (Hoschouer et al., 2010; Detloff et al., 2013; Yang et al., 2014; Carbajal et al., 2020; Brown et al., 2022). Despite closely mimicking human SCI trauma scenario, contusion studies face limitations due to variable damage severity across experiments. In contrast, lateral hemisection surgery provides reproducible anatomical damage and locomotor outcomes (Ballermann and Fouad, 2006; Filli et al., 2011; Takeoka et al., 2014; Liu et al., 2017), facilitating mechanistic studies. Dr. Hulsebosch pioneered the adaptation of T13 lateral hemisection model three decades ago to examine thermal and mechanical allodynia and reported bilateral hypersensitivity in both fore- and hindpaws (Christensen et al., 1996). Yet BSS typically manifests as contralateral below-level allodynia (Schott, 1993; Loch-Wilkinson et al., 2018); ipsilateral pain, when present, is transient and resolves quickly (Riddoch, 1938; Garcin et al., 1968; Pagni, 1998). We reasoned that T13 injuries, being adjacent to the lumbar spinal cord, potentially caused widespread inflammation and microglial activation in the lumbar region (Li et al., 2020; Brennan et al., 2022), complicating allodynia readouts (Guan et al., 2015). Additionally, the observed behaviors from her study has yet validated in the anatomical analysis. To address these issues, we made two major changes in our current study. First, we performed lateral hemisection at the T8 level, ∼7 mm away from the lumbar region in mice, to minimize surgically induced inflammation affecting the lumbar areas. Second, we conducted a viral-based ascending spinal circuit investigation to further validate the sensory deficits in this model. Together, our mouse data recapitulate the human pattern, validating the T8 hemisection as an appropriate tool for investigating asymmetric chronic dysesthesia.

Thermal changes after hemisection diverge between humans and rodents. BSS patients show normal ipsilateral thermal sensation but reduced contralateral sensitivity (Park et al., 2015; Nelson et al., 2020; Hoebee et al., 2022), whereas prior rodent studies report bilateral hyperalgesia (Christensen et al., 1996; Detloff et al., 2013; Martini et al., 2016; Brown et al., 2021; Lin et al., 2022). In this study, we paired multiple sensory tests with anatomical tracing to interpret thermal changes after T8 hemisection. At 4 wpi, dry ice stimulation was unchanged, whereas the Hargreaves test revealed contralateral hyperalgesia—suggesting heightened heat sensitivity on the contralateral side but none ipsilaterally. Paw withdrawal latencies can be driven by spinal reflexes (Barik et al., 2018), so withdrawal-based assays alone may misrepresent supraspinal thermal perception. Intradermal capsaicin (TRPV1 agonist) greatly reduced nocifensive licking in both paws, implying bilateral hyposensitivity. Circuit tracing offers distinct mechanisms: loss of the ipsilateral capsaicin response aligns with disruption of the ipsilateral SPB pathway, whereas contralateral hyposensitivity reflects STT-tract interruption. On a 52°C hot plate, 85% of SCI mice adopted a leaning posture, and 71% relied on the contralateral paw, indicating reduced heat sensitivity in that limb, while the ipsilateral paw remained normal. Hot plate escape, which requires supraspinal processing (Vierck and Light, 2000; Vierck et al., 2004, 2000), thus confirms a Brown–Séquard-like asymmetry: preserved ipsilateral and diminished contralateral heat sensitivity. Collectively, these findings show that no single assay suffices; converging evidence from multiple tests, interpreted in the context of anatomical circuitry, is essential for accurately characterizing sensory deficits after SCI.

For translational purposes, our results may provide valuable insights that could enhance current diagnostic and care standards for patients with SCI. Chronic neuropathic dysesthesias, characterized by a mixture of excruciatingly unpleasant sensations, are exceedingly difficult to manage and greatly detract from quality of life (Beric et al., 1988; Finnerup et al., 2001). If it were possible to predict which patients are at risk for developing neuropathic dysesthesias, preemptive treatment could be initiated and prepared. In our study, mice with T8 hemisection developed chronic contralateral dysesthesias (Fig. 3*A*–*I*) and, strikingly, a concomitant reduction in noxious responsiveness in the same paw (Fig. 2*B,E*). A similar dissociation—loss of nociception coupled with neuropathic pain—has been documented in SCI patients (Levitan et al., 2015), suggesting that diminished noxious perception may predispose to dysesthesia. Additionally, recent clinical studies have indicated that individuals with SCI are at an increased risk of self-harm compared with the general population over the past three decades in the USA (Cao et al., 2014; Kennedy and Garmon-Jones, 2017). In our studies, we observed a high rate of self-injury behavior (62.1%) in SCI mice compared with intact mice (0%). Our results echo clinical findings and highlight the urgent need for psychological and psychiatric assessment and intervention for individuals with SCI.

For research purpose, the T8 lateral hemisection SCI model offers a unique opportunity to examine neuroplasticity of severed tracts or the roles of potential alternative synaptic pathways that contribute to sensory deficits observed. Particularly, the mechanisms underlying the development of chronic neuropathic pain post-SCI are not well understood (Watson and Sandroni, 2016). Our findings indicate that chronic neuropathic pain and self-injurious behaviors manifest only in the contralateral hindpaw, providing a robust within-subject comparison to study neuroplastic changes. This insight could enhance therapeutic approaches, including drug screening and gene therapy. Additionally, restoring light or pleasant touch, a critical yet understudied patient priority, may benefit from strategies promoting axon regeneration (Liu et al., 2010, 2017; Anderson et al., 2018; Brennan et al., 2022; Squair et al., 2023). Given that spinal projection neurons involved in light touch are well characterized (Abraira and Ginty, 2013; Choi et al., 2020; Turecek et al., 2022), it may be feasible to test if enhancing axon regeneration in these neurons could improve light-touch sensitivity in the T8 lateral hemisection SCI mouse model.

In conclusion, we characterized and validated a mouse lateral hemisection SCI model that closely mirrors the key features and syndromes of BSS. By comparing clinical conditions of BSS with our preclinical observations from multiple behavioral assays and tracing major ascending tracts, we established a robust SCI mouse model that specifically mimics sensory deficits. Future studies using this model are essential to directly test the underlying mechanisms of sensory deficits and to explore therapeutic approaches.

### Limitations of the study

Our study has some limitations. First, our model is unlikely to fully replicate the wide range of sensory changes and deficits observed in patients with SCI, given the complexity and heterogeneity of SCI severity and the diversity of sensory and behavioral phenotypes. Second, our sensory outcome measures primarily focused on evoked and spontaneous cutaneous and musculoskeletal hypersensitivity or hyposensitivity in the hindpaws, aiming to mimic features of BSS. However, sensory deficits in BSS patients often extend beyond the foot and may spread below the injury site, which we did not evaluate in this study. Future research should assess sensory features across different body regions, both above and below the injury.

## Data Availability

This study did not generate new unique reagents. Datasets, custom algorithms, and unique code supporting the current study are available from the corresponding author on request.
